# Emulating the MERINO randomised control trial using data from an observational cohort and trial of rapid diagnostic (BSI-FOO)

**DOI:** 10.1371/journal.pone.0268807

**Published:** 2022-05-20

**Authors:** Rebecca N. Evans, Jessica Harris, Chris A. Rogers, Alasdair MacGowan

**Affiliations:** 1 Bristol Trials Centre, Bristol Medical School, University of Bristol, Bristol, United Kingdom; 2 Infection Sciences, Pathology, North Bristol NHS Trust, Bristol, United Kingdom; University of Ottawa, CANADA

## Abstract

**Objective:**

The aim of this study was to emulate the MERINO trial of piperacillin-tazobactam vs meropenem for the definitive treatment of bloodstream infection (BSI) caused by ceftriaxone-nonsusceptible *E coli* or Klebsiella spp.

**Methods:**

Data from an observational study of BSI and a randomised controlled trial of a rapid diagnostic in BSI were used to emulate the MERINO trial. The primary outcome of the emulated trial was 28-day mortality after blood culture. Outcomes were compared using logistic regression adjusted for propensity score for emulated intervention.

**Results:**

Of the 6,371 observational study and RCT participants, 1,968 had a bloodstream infection *with E*. *coli* or Klebsiella spp. of which 121 met the eligibility criteria. In the emulated trial, a total of 14/82 patients (17.1%) allocated to piperacillin-tazobactam met the primary outcome compared with 6/39 (15.4%) in the meropenem group (unadjusted odds ratio 1.13 (95% CI 0.40 to 3.21)). After adjustment for propensity score, the odds ratio increased to 1.31 (95% CI 0.40 to 4.26). This difference is in the same direction but of a smaller magnitudethan observed in the MERINO trial, where 30-day mortality was met by 23/187 patients (12.3%) in the piperacillin-tazobactam and 7/191 (3.7%) in the meropenem group (unadjusted odds ratio of 3.69 (95% CI 1.48 to 10.41)).

**Conclusions:**

The mortality rate in an emulated trial population was more than double the mortality rate in the MERINO trial. The methodology used attempts to address the concern that previous results could be explained by biases such as selection bias and uncontrolled confounding and provides information on how a trial such as the MERINO trial may have performed in the NHS.

## Introduction

Extended-spectrum β-lactamase (ESBL) producing bacteria are a frequent cause of bloodstream infection (BSI). Carbapenems have been regarded as the antibiotic of choice for treatment of infections caused by ESBL producers [1]. However, it has been shown that increased use of carbapenems is associated with increased incidence of carbapenem resistant Enterobacteriaceae [2]. Alternative treatments are needed to help contain the spread and frequency of carbapenem resistance. β-Lactam/β-lactamase inhibitor (BLBLI) combination antibiotics, such as piperacillin-tazobactam, have been considered a carbapenem-sparing option for treatment of ESBL producers [3, 4]. There have been a number of observational studies that have shown that BLBLIs are an effective treatment for infections caused by ESBL producers [5–9], and recent reviews have shown this to differ depending on the infection severity [10, 11]. However, observational analyses are subject to bias and results from a recent randomised controlled trial (RCT), the MERINO trial [12], were not consistent with results published in these observational studies. The MERINO trial is a recent international RCT to determine whether definitive therapy with piperacillin-tazobactam is noninferior to meropenem in patients with BSI caused by ceftriaxone-non-susceptible *E*.*coli* or *K*.*pneumoniae* [12]. A noninferiority margin of 5% was used. The MERINO trial concluded that definitive treatment with piperacillin-tazobactam did not result in noninferior 30-day mortality compared to definitive treatment with meropenem.

An RCT is considered the gold standard design in clinical research, however, they are not always financially or ethically feasible to conduct. Therefore, an observational approach is often used but these studies are subject to bias and confounding. Emulating a target trial is an approach designed to “mimic” trial practice using observational data and if successful, should yield similar results [13].

The aim of this study was to use data from an observational study and a trial of a rapid diagnostic to emulate the MERINO trial to explore whether an emulated trial would yield consistent results using data from the UK.

## Methods

### Study design

Patient-level data from the two studies performed as part of the BSI-FOO Programme [14, 15] were used to emulate the MERINO trial eligibility criteria, treatment strategy, and statistical analysis. The two studies were part of the same NIHR research programme and data collection was similar. Therefore, to maximise the potential sample size of the emulated trial, data from both studies were used. One was the BSI-FOO observational study and the other the RAPIDO RCT (trial registration ISRCTN97107018). The BSI-FOO observational study was a multicentre cohort study of 1,903 hospitalised patients with a BSI across seven NHS acute hospital trusts in England and Wales conducted between November 2010 and May 2012 with the primary aim of identifying modifiable risk factors for 28-day mortality. Adults (≥18 years old) receiving in-patient NHS hospital care and having a clinically significant BSI caused by six key pathogens: 1) methicillin-resistant Staphyloccos aureus (MRSA); 2) methicillin-susceptible S. aureus (MSSA); 3) non-Extended-spectrum beta-lactamase (ESBL)-producing Escherichi coli; 4) any ESBL-producing member of the family Enterobacteriales; 5) Pseudomonas aeruginosa; 6) any species of Candida, were included. RAPIDO was a multicentre open parallel group (1:1) RCT comparing two approaches to the identification of the causative microorganism(s) of BSI in hospitalised adult patients. The RAPIDO trial took place in seven NHS acute hospital trusts in England and Wales between July 2012 and August 2014. Date and time 0 was the date and time of first positive blood sample confirming BSI in both the observational study and the RCT. Full details of the inclusion criteria and study designs are given in the study results publications [14, 15].

The research programme on which this work is based was approved by Southwest Research Ethics Committee (10/HO102/51). The National Information Governance Board approved the use of routinely-collected patient data without specific consent for the BSI-FOO observational study and collection of full data for patients who died before being approached for consent in RAPIDO. North Bristol NHS Trust acted as Sponsor.

### Study population

The MERINO trial inclusion/exclusion criteria were applied to BSI-FOO and RAPIDO participants (Table 1). Patients were included if they had a BSI with *E*. *coli* or *Klebsiella* spp. that was resistant to Ceftriaxone and/or Cefotaxime and susceptible to both meropenem and piperacillin-tazobactam and treatment was started within 72 hours of blood culture (date and time 0 for BSI-FOO and RAPIDO). Patients that would have otherwise been eligible but did not start any of the study drugs (meropenem or piperacillin-tazobactam) within the 72 hours window were considered ineligible and excluded from the emulated trial population. All participants were aged 18 years and over. Informed consent was not required for the BSI-FOO observational study and RAPIDO patients who died before being approached for consent, so it was not possible to replicate consent for this population, but all surviving RAPIDO participants provided written informed consent to join the trial. Exclusion criteria are given in Table 1.

**Table 1:** Population, intervention, comparison, outcome (PICO) table.

PICO component		MERINO trial	Emulated trial (BSI FOO & RAPIDO)
Patient/population	Inclusion	Bloodstream infection with *E*. *coli* or Klebsiella spp. with proven non-susceptibility to third generation cephalosporins and susceptibility to meropenem and piperacillin-tazobactam	• ESBL producing *E*.*coli* AND Klebsiella–ESBL • Resistant to Ceftriaxone or Cefotaxime • Susceptible to Meropenem AND piperacillin-tazobactam
		No more than 72 hours has elapsed since the first positive blood culture collection	• Start treatment within 72 hours of blood culture • Received either Meropenem or piperacillin-tazobactam in that window
		Patient is aged 18 years and over	All BSI-FOO and RAPIDO participants
		The patient or approved proxy is able to provide informed consent	All BSI-FOO and RAPIDO participants
	Exclusion	Patient not expected to survive more than 4 days	Not applied
		Patient allergic to a penicillin or a carbapenem	Assume if in receipt of drug then no known allergy
		Patient with significant polymicrobial bacteraemia	Polymicrobial infections
		Previously enrolled	Repeat episodes
		Treatment is not with the intent to cure the infection (that is, palliative care is an exclusion).	End of care pathway excluded in BSI-FOO and RAPIDO
		Pregnancy or breast-feeding	Not applied
		Use of concomitant antimicrobials in the first 4 days after enrolment with known activity against Gram-negative bacilli (except trimethoprim/sulfamethoxazole may be continued as Pneumocystis prophylaxis).	Not applied
Intervention	Piperacillin-tazobactam	4.5g administered every 6 hours intravenously.	As prescribed*
Comparison	Meropenem	1g will be administered every 8 hours intravenously.	As prescribed**
		Each dose will be given over 30 minutes. The study drug is to be administered for a minimum of 4 days and can be given for as long as 14 days. The total duration of therapy will be determined by the treating clinician. Dose adjustment for renal impairment will be made	
Outcome	Follow up	Starts at assignment to intervention and ends at death or 30 days.	Start on date/time of first prescribed study drug
	Primary outcome	30-day mortality	25-day mortality

* Dose: 4.5g (99%), 2.25g (1%). Frequency: 3/day (68%), 2/day (13%), 1/day (1%), stat (17%).

** Dose: 1g (74%), 2g (3%), 0.5g (23%). Frequency: 3/day (54%), 2/day (23%), 1/day (5%), stat (18%).

**Abbreviations:** PICO = Population, intervention, comparison, outcome.

### Intervention

The trial interventions in the MERINO trial were treatment with piperacillin-tazobactam or meropenem. These were to be administered for a minimum of 4 days and maximum of 14 days, with duration determined by the treating clinician. In the emulated trial population patients were assigned to a “emulated intervention” based on their treatment timeline and allocated to the first study drug received. The start of follow-up was defined as the date in which the patient started their first dose of their assigned intervention.

### Outcome measures

The primary outcome of the MERINO trial was all-cause mortality at 30 days after randomisation. It was not possible to analyse 30-day mortality for the emulated trial as follow-up in BSI-FOO and RAPIDO was limited to 28 days, where start of follow-up was defined as the date the blood sample was taken. All patients started their emulated intervention within 3 days of blood sample; therefore, 25-day mortality was analysed to ensure full follow-up was available for all patients.

### Statistical analyses

The MERINO trial analysis population was defined as any randomised participant receiving at least 1 dose of the allocated drug. This was supported by an analysis of the per-protocol population. By definition, in the emulated trial population, all participants received at least one dose of allocated drug and were therefore included in the primary analysis population. We did not emulate the per-protocol analysis as few participants received allocated treatment for the required four days.

Continuous data were summarised using mean and standard deviation (or median and interquartile range (IQR) if distributions were skewed) and categorical data as numbers and percentages. Demographics, comorbidities and medical history were summarised by emulated intervention. Standardised mean differences were calculated to quantify imbalances in baseline characteristics by the treatment group [16]. Mortality over 25-days was summarised by emulated intervention using inverse probability weighted survival curves (weighted according to the inverse probability of treatment received, see below for further details of propensity score) to show adjusted survival graphically [17].

To emulate the trial analyses, absolute risk differences were calculated using generalised linear models. As the emulated trial was not randomised, potential confounding factors needed to be accounted for in the analysis. Propensity score models were developed using a logistic regression model with emulated intervention as the outcome. Variables included in the propensity score model were age and sex and any potential confounders based on clinician expertise. These were specified *a priori*. Factors included in the propensity score model were: centre, age, sex, temperature at time 0, neutrophil count on day 0 or closest, systolic blood pressure on day 0 or closest, on IV fluids at day, on ventilation at day 0, cerebrovascular disease, Charlson score and source of infection. The number of participants and deaths in each emulated intervention group was examined within strata defined by propensity score quantiles. Participants in strata for which there were no participants or deaths in either group were excluded to ensure the analyses were restricted to participants eligible to receive either treatment strategy, ensuring the assumption of positivity was met.

Convergence was not achieved when fitting an adjusted generalised linear model and therefore outcomes were compared using logistic regression adjusted for the propensity score. Unadjusted odds ratios were calculated for the MERINO trial to provide a comparison. Propensity scores were modelled using restricted cubic splines with 3 knots at 10^th^, 50^th^ and 90^th^ percentiles to capture potential non-linear associations with the outcome. Missing values were imputed with age- and sex-adjusted averages.

Three sensitivity analyses were carried out: (a) imputing missing categorical values with worst case values i.e. disease present; (b) propensity score model using restricted cubic splines at 25^th^, 50^th^ and 75^th^ percentiles to assess the robustness of the results to the location of knots; (c) excluding participants that switch to the other intervention during follow-up.

Model fit was assessed using standard methods. Meropenem was the reference group in all analyses. Results are reported as effect estimates with 95% confidence intervals (CI). In all tables missing data are described in footnotes.

All analyses were performed in Stata version 16.0 (StataCorp, LP, College Station, TX, US).

## Results

Of the 6,371 BSI-FOO and RAPIDO participants, 1,968 had a BSI with E. coli or *Klebsiella* spp. of which 163 had proven non-susceptibility to cephalosporins and proven susceptibility to meropenem and piperacillin-tazobactam. Of these, 34 were not in receipt of meropenem or piperacillin-tazobactam within 72 hours of blood culture. Of the remaining 129 participants, 4 repeat episodes and 4 polymicrobial infections were excluded, thus 121 met the eligibility criteria (S1 Fig in S1 File) and were included in the analysis population. No observations were excluded based on propensity scores/confounders.

### Intervention

Of the 121 participants who met the emulated trial eligibility criteria, 82 were assigned to piperacillin-tazobactam and 39 to meropenem, according to their first study drug received. The median time to receipt of study drug was longer in the meropenem group (38 hours (IQR 8, 54)) compared to the piperacillin-tazobactam group (6 hours (IQR 0, 19)) and duration of allocated treatment was also longer in the meropenem group (7 days (IQR 4, 8) vs. 3 days (IQR 2, 5)). Of those allocated to meropenem, 31/39 (79.5%) were in receipt of their study drug for four days (MERINO trial per-protocol) and of those allocated to piperacillin-tazobactam, 38/82 (46.3%) were in receipt of their study drug for four days. After the first dose of emulated intervention, 1/39 (2.6%) participants allocated to meropenem switched to piperacillin-tazobactam, conversely, 39/82 (47.6%) switched from piperacillin-tazobactam to meropenem (S1 Table in S1 File).

### Demographics

Demographic characteristics and medical history are shown by emulated intervention in Table 2 and for the MERINO trial vs. emulated trial in S2 Table in S1 File and inverse probability weighted in S3 Table in S1 File. Overall, the participant characteristics were similar to the MERINO population with the exception of Charlson comorbidity index which was slightly higher (3.0 vs. 2.0) and moderate-severe renal dysfunction which was present in a higher proportion of participants (61% vs. 16%) in the emulated trial population.

**Table 2:** Baseline characteristics of patients in the emulated trial population, by emulated intervention.

		Meropenem (n = 39)		Piperacillin-Tazobactam (n = 82)		SMD (M-PT)	Overall (n = 121)	
		n	%	n	%		n	%
**Patient measures**								
Age	Median (IQR)	70.0 (54.0, 82.0)		74.5 (63.0, 84.0)		-0.29	73.0 (61.0, 83.0)	
Male		15/39 (38.5%)		46/82 (56.1%)		0.36	61/121 (50.4%)	
Body Mass Index ^a^	Mean (SD)	25.3 (9.2)		24.7 (5.0)		0.07	24.9 (6.6)	
**Patient medical history**								
Chemotherapy in month before date 0		1/39 (2.6%)		15/82 (18.3%)		0.53	16/121 (13.2%)	
Any tumour within last 5 years		12/39 (30.8%)		29/82 (35.4%)		0.10	41/121 (33.9%)	
Surgery requiring overnight stay within 7 days before date 0		2/39 (5.1%)		3/82 (3.7%)		0.07	5/121 (4.1%)	
Burn requiring admission within 7 days before date 0		0/32 (0.0%)		0/59 (0.0%)		-	0/91 (0.0%)	
Cardiac arrest within 7 days before date 0		0/39 (0.0%)		0/82 (0.0%)		-	0/121 (0.0%)	
Renal support within 7 days before date 0		2/39 (5.1%)		2/82 (2.4%)		0.14	4/121 (3.3%)	
Myocardial infarction within 7 days before date 0		3/39 (7.7%)		9/82 (11.0%)		0.11	12/121 (9.9%)	
**Infection severity measures**								
Temperature (°C) at time 0 ^b^	Median (IQR)	38.4 (38.0, 39.0)		38.0 (37.1, 38.5)		0.48	38.2 (37.4, 38.7)	
INR ^c^	Median (IQR)	1.3 (1.2, 2.8)		1.1 (1.1,.)		0.04	1.2 (1.1, 1.5)	
eGFR (mL/min/1.73m^2^) ^d^	Median (IQR)	53.0 (31.7, 81.0)		49.0 (29.0, 77.4)		0.12	49.5 (29.0, 79.0)	
Neutrophil count at day 0 or closest (x10^9^/L) ^e^	Median (IQR)	10.3 (6.9, 13.3)		11.2 (4.9, 16.2)		-0.03	10.8 (5.1, 15.3)	
Systolic BP at day 0 or closest (mmHg) ^f^	Mean (SD)	129.6 (28.8)		116.3 (29.2)		0.46	120.7 (29.6)	
On IV fluids at day 0		16/39 (41.0%)		37/82 (45.1%)		0.08	53/121 (43.8%)	
On ventilation at day 0		6/39 (15.4%)		4/82 (4.9%)		0.35	10/121 (8.3%)	
On vasopressor drugs at day 0		3/39 (7.7%)		1/82 (1.2%)		0.32	4/121 (3.3%)	
Systemic corticosteroids in last 24 hours		5/39 (12.8%)		9/82 (11.0%)		0.06	14/121 (11.6%)	
EWS score nearest to day 0^g^		4.0 (2.0, 6.0)		2.0 (1.0, 3.5)		0.62	2.0 (1.0, 4.0)	
**Patient comorbidities at date 0**								
Congestive heart failure		4/39 (10.3%)		10/82 (12.2%)		0.06	14/121 (11.6%)	
Peripheral vascular disease		4/39 (10.3%)		9/82 (11.0%)		0.02	13/121 (10.7%)	
Cerebrovascular disease		10/39 (25.6%)		20/82 (24.4%)		0.03	30/121 (24.8%)	
Hemiplegia		0/39 (0.0%)		5/82 (6.1%)		0.36	5/121 (4.1%)	
Dementia		5/39 (12.8%)		10/82 (12.2%)		0.02	15/121 (12.4%)	
COPD		6/39 (15.4%)		11/82 (13.4%)		0.06	17/121 (14.0%)	
Connective tissue disease		2/39 (5.1%)		6/82 (7.3%)		0.09	8/121 (6.6%)	
Peptic ulcer disease		4/39 (10.3%)		6/82 (7.3%)		0.10	10/121 (8.3%)	
Ascites		1/39 (2.6%)		3/82 (3.7%)		0.06	4/121 (3.3%)	
Diabetes:								
None		29/39 (74.4%)		57/82 (69.5%)		0.11	86/121 (71.1%)	
Without organ damage		8/39 (20.5%)		16/82 (19.5%)		0.03	24/121 (19.8%)	
With organ damage		2/39 (5.1%)		9/82 (11.0%)		0.22	11/121 (9.1%)	
Child-Pugh score ^h^	Median (IQR)	6.0 (5.0, 8.0)		6.0 (6.0, 9.0)		-0.36	6.0 (6.0, 8.0)	
Charlson score ^i^	Median (IQR)	4.0 (2.0, 5.0)		3.0 (2.0, 4.0)		-0.01	3.0 (2.0, 4.5)	
Abscess at time 0		0/32 (0.0%)		2/59 (3.4%)		0.26	2/91 (2.2%)	
Infected foreign body at time 0		1/32 (3.1%)		0/59 (0.0%)		-	1/91 (1.1%)	
Surgical prosthesis time 0		0/32 (0.0%)		1/59 (1.7%)		0.19	1/91 (1.1%)	
**Source of infection**								
Bone and joint		1/39 (2.6%)		0/82 (0.0%)		0.57	1/121 (0.8%)	
Gastrointestinal system		6/39 (15.4%)		10/82 (12.2%)			16/121 (13.2%)	
Line infection–central venous line		1/39 (2.6%)		1/82 (1.2%)			2/121 (1.7%)	
Lower respiratory tract		1/39 (2.6%)		1/82 (1.2%)			2/121 (1.7%)	
Reproductive tract		1/39 (2.6%)		0/82 (0.0%)			1/121 (0.8%)	
Skin and soft tissue		1/39 (2.6%)		0/82 (0.0%)			1/121 (0.8%)	
Surgical site infection		0/39 (0.0%)		1/82 (1.2%)			1/121 (0.8%)	
Systemic Infection		1/39 (2.6%)		0/82 (0.0%)			1/121 (0.8%)	
Urinary tract infection		20/39 (51.3%)		46/82 (56.1%)			66/121 (54.5%)	
Site uncertain		7/39 (17.9%)		23/82 (28.0%)			30/121 (24.8%)	
**Lines and catheters**								
Central line present at time 0		4/32 (12.5%)		11/59 (18.6%)		0.17	15/91 (16.5%)	
Peripheral line present at time 0		15/32 (46.9%)		34/59 (57.6%)		0.22	49/91 (53.8%)	
Urinary catheter present at time 0		7/32 (21.9%)		21/59 (35.6%)		0.31	28/91 (30.8%)	
**Organisational factors**								
Centre:								
A		1/39 (2.6%)		8/82 (9.8%)		0.54	9/121 (7.4%)	
B		8/39 (20.5%)		14/82 (17.1%)			22/121 (18.2%)	
C		13/39 (33.3%)		31/82 (37.8%)			44/121 (36.4%)	
D		9/39 (23.1%)		10/82 (12.2%)			19/121 (15.7%)	
E		5/39 (12.8%)		17/82 (20.7%)			22/121 (18.2%)	
F		1/39 (2.6%)		0/82 (0.0%)			1/121 (0.8%)	
G		2/39 (5.1%)		2/82 (2.4%)			4/121 (3.3%)	
Ward specialty on day 0:								
Medicine		20/39 (51.3%)		52/82 (63.4%)		0.37	72/121 (59.5%)	
Critical care		4/39 (10.3%)		6/82 (7.3%)			10/121 (8.3%)	
Major surgery		12/39 (30.8%)		16/82 (19.5%)			28/121 (23.1%)	
Minor surgery		0/39 (0.0%)		2/82 (2.4%)			2/121 (1.7%)	
Other		3/39 (7.7%)		6/82 (7.3%)			9/121 (7.4%)	

**Notes:** Date and time 0 = date/time of sampling for blood culture.

^a^ Data missing for 78 patients (24 Meropenem, 54 Piperacillin-Tazobactam).

^b^ Data missing for 3 patients (3 Meropenem, 0 Piperacillin-Tazobactam).

^c^ Data missing for 56 patients (19 Meropenem, 37 Piperacillin-Tazobactam).

^d^ Data missing for 3 patients (2 Meropenem, 1 Piperacillin-Tazobactam).

^e^ Data missing for 3 patients (1 Meropenem, 2 Piperacillin-Tazobactam).

^f^ Data missing for 12 patients (3 Meropenem, 9 Piperacillin-Tazobactam).

^g^ Data missing for 76 patients (26 Meropenem, 50 Piperacillin-Tazobactam).

^h^ Data missing for 80 patients (26 Meropenem, 54 Piperacillin-Tazobactam).

^i^ Data missing for 29 patients (10 Meropenem, 19 Piperacillin-Tazobactam).

**Abbreviations:** SMD = Standardised mean difference, IQR = Interquartile range, SD = Standard deviation, INR = International normalised ratio, eGFR = Estimated glomerular filtration rate, BP = Blood pressure, IV = Intravenous, EWS = Early warning score, COPD = Chronic obstructive pulmonary disease.

### Primary outcome

**Fig 1:**
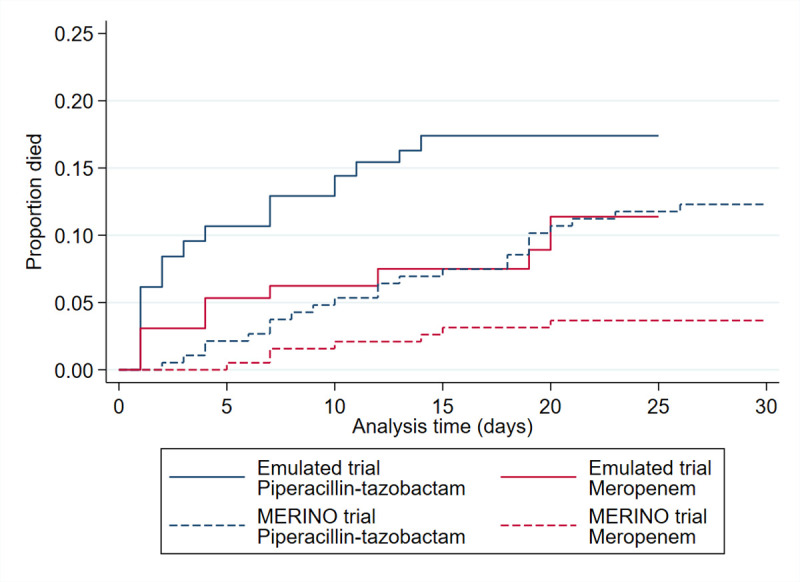
Inverse probability weighted Kaplan-Meier, by emulated intervention.

The overall 25-day mortality rate was 20/121 (16.5%) compared to 30/378 (7.9%) 30-day mortality in the MERINO trial population [12]. Inverse probability weighted Kaplan-Meier curves displaying time to death according to emulated intervention group are shown in Fig 1. In this emulated trial, a total of 14/82 participants (17.1%) allocated to piperacillin-tazobactam met the primary outcome of all-cause mortality at 25 days compared with 6/39 (15.4%) in the meropenem group (risk difference 1.7%, 95% CI -12.26 to 15.64). The corresponding unadjusted odds ratio is 1.13 (95% CI 0.40 to 3.21). After adjustment for propensity score, the odds ratio increased to 1.31 (95% CI 0.40 to 4.26). Sensitivity analysis gave similar results (Table 3). These differences are lower than observed in the MERINO trial, where 30-day mortality was met by 23/187 participants (12.3%) in the piperacillin-tazobactam and 7/191 (3.7%) in the meropenem group with an unadjusted risk difference 8.6 (95% CI 3.0 to 14.5) and odds ratio of 3.69 (95% CI 1.48 to 10.41). Sensitivity analyses gave similar results.

**Table 3:** Primary analysis: 25-day mortality.

Population	Meropenem		Piperacillin-Tazobactam		N	Estimate
	n	%	n	%		RD/OR (95% CI)
**MERINO TRIAL (30-day mortality)**	**7/191**	**3.7%**	**23/187**	**12.3%**		
Unadjusted risk difference					278	RD = 8.6 (95% CI 3.4 to 14.5)
Unadjusted odds ratio					278	OR = 3.7 (95% CI 1.5 to 10.4)
**EMULATED TRIAL (25-day mortality)**	**6/39**	**15.4%**	**14/82**	**17.1%**		
Unadjusted risk difference					121	RD = 1.69 (95% CI -12.26 to 15.64)
Unadjusted odds ratio					121	OR = 1.13 (95% CI 0.40 to 3.21)
Propensity score adjusted *					121	OR = 1.31 (95% CI 0.40 to 4.26)
Sensitivity analysis 1 **					121	OR = 1.38 (95% CI 0.43 to 4.45)
Sensitivity analysis 2 ***					121	OR = 1.29 (95% CI 0.40 to 4.17)
Sensitivity analysis 3 ****	6/38	15.8%	8/43	18.6%	81	OR = 1.61 (95% CI 0.38 to 6.73)

* Propensity score adjustment. Propensity score calculated using centre, age, sex, chemotherapy in month before date 0, temperature at time 0, neutrophil count at day 0, SBP, on IV fluids, on ventilation, Cerebrovascular disease, Charlson score, source of infection. Modified Charlson score, temperature at time 0, neutrophil count and SBP imputed using conditional mean imputation. Propensity score modelled using restricted cubic splines with 3 knots at 10^th^, 50^th^ and 90^th^ percentiles.

** SA1: Adjusted for propensity score: Charlson score imputed using worst case scenario (liver disease present and moderate/severe kidney disease).

*** SA2: Adjusted for propensity score: Propensity score modelled using restricted cubic splines with 3 knots at 25^th^, 50^th^ and 75^th^ percentiles.

**** SA3: Excluding participants that switch to the other intervention during follow-up.

**Abbreviations:** RD = Risk difference, OR = Odds ratio, SBP = Systolic blood pressure, IV = Intravenous.

## Discussion

The overall mortality rate in the emulated trial was more than double the mortality rate in the MERINO trial but similar to mortality rates reported in other observational studies [5, 6]. Although the effect size was smaller in the emulated trial (OR = 1.31 compared to 3.7 in the MERINO trial), the 95% confidence interval for the emulated trial estimate (95% CI 0.4 to 4.26) includes the estimated odds ratio from the MERINO trial which suggests potential consistency given the smaller sample size in this study.

There are several differences in the study design and population characteristics that could explain the lower mortality rates observed in the MERINO trial compared to the emulated trial. Firstly, there are differences in the demographics, comorbidities and severity of illness between the emulated trial population and the MERINO trial population. Secondly, the process of obtaining patient consent in MERINO could result in the sickest patients not being captured. Consent was not required for the BSI-FOO observational study and therefore all eligible patients were included. In addition, the exclusion of individuals who were deemed unlikely to survive beyond 96 hours in MERINO may have also resulted in the sicker patients who would otherwise be eligible for the trial being excluded which could lead to an underestimation of the true mortality rate. This was acknowledged by the authors as a limitation of the study. We did not impose the 96-hour restriction in the trial emulation as this could have introduced survival bias. Finally, over half of the infections in the MERINO trial were urinary tract which are known to be more responsive to treatment. The treatment effect may differ across different levels of infection severity/presence of comorbidities e.g. piperacillin-tazobactam may be inferior to carbapenems in patients with severe infections but non-inferior in less severe infections such as urinary tract infections [10, 11], however we did not have a large enough sample size to explore this. Further research is required to investigate this, but this may in part explain the conflicting results published in the MERINO trial to other observational studies where the populations and severity of illness are likely to differ.

### Strengths and limitations

One of the strengths of this study is the trial emulation approach used in the design and analysis. Trial emulation using observational data ensures that eligibility criteria and assumptions are explicit before analysis and minimises common biases that arise in observational data analyses. In addition, a number of the published observational studies exclude patients who are not in receipt of either intervention for >48hours [7–9], meaning patients who die within 48 hours of receipt treatment are excluded from the population which gives potential for introducing survival bias. Applying trial emulation methods enabled us to include all patients who would be eligible for a trial, without using data after start of follow-up in the inclusion/exclusion criteria, therefore minimising survival bias. In addition, no UK patients were recruited to the MERINO trial, so applying trial emulation methods to UK data provides information on how a trial such as the MERINO trial may have performed in the NHS and the addition of UK data to the literature adds to the generalisability of the currently published work.

There are several limitations to this study. First, it was not possible to emulate all elements of the MERINO trial. The MERINO trial had 30-day follow-up, we did not have 30-day follow-up for all BSI-FOO and RAPIDO participants and therefore we analysed 25-day mortality. However, this is unlikely to have a significant impact as later deaths are less unlikely to be a result of treatment. Upon examination of the Kaplan-Meier curves, there is a larger difference in mortality rate up to 14 days. We did not formally compare mortality rates at 14 days due to the small number of events and lack of statistical power, however we feel that 14-day mortality may be a more clinically meaningful outcome for studies investigating mortality in BSI as it is the time period most reflective of death attributable to suboptimal therapy. In addition, we did not explore other outcomes other than mortality as this was beyond the scope of the trial emulation, however other outcomes such as composite outcome of treatment failure and mortality may merit analysis in future research.

Secondly, it was not possible to emulate the per-protocol analysis fully as few participants received allocated treatment for four days, and restricting analyses to those who are in receipt for four or more days would introduce immortal time bias (bias induced by a period of follow-up during which, by design, the outcome cannot occur). Our approach made it hard to attribute differences in the intention-to-treat analysis because some participants received both treatments with many of the participants assigned to piperacillin-tazobactam swapping to meropenem leading to contamination of drug exposure. This compromises our ability to draw any firm conclusions from this study. We excluded participants who switched to the other intervention during follow-up in a sensitivity analysis and this gave similar results, although it is worth noting that this sensitivity analysis had a smaller sample size, and this analysis is likely to be subject to selection bias as switching is usually related to prognosis. In addition, participants’ empirical treatment and treatment pathways other than the “allocated” intervention were not controlled for so any observed differences could be attributed to the effects of empirical therapy or treatments received after classification of “trial drug”. There were also differences in the time to receipt of “allocated” treatment between the two groups.

Finally, it was not possible to emulate blinding, so the validity of our estimates depends on the assumption that all confounding factors were correctly adjusted for. We allowed for differences in baseline characteristics by adjusting for propensity score. However, due to the retrospective nature of the study we were only able to control for variables that had been collected and there is the risk that unmeasured confounders may impact the results.

In summary, the mortality rate in an emulated trial population was more than double the mortality rate in the MERINO trial and the difference between mortality rates in piperacillin-tazobactam and meropenem was weaker but the direction of effect was consistent with the MERINO trial. Our findings suggest that the discrepancies between the MERINO trial and observational studies estimates could be partly explained by differences in the populations and also due to the bias that arises from observational studies e.g. survival bias and bias from unmeasured or uncontrolled confounding. This methodology attempts to address the concern that previous results could be explained by such biases and provides information on how a trial such as the MERINO trial may have performed in the NHS.

## Supporting information

S1 File Emulating the MERINO trial—Supplementary material. (PDF)
