# Supplementary material for: Emulating the MERINO randomised control trial using data from an observational cohort and trial of rapid diagnostic (BSI-FOO)
Source: PLoS One. 2022 May 20;17(5):e0268807. doi: 10.1371/journal.pone.0268807 (PMC9122188; doi:10.1371/journal.pone.0268807)
Supplement: S1 File — (PDF) [file pone.0268807.s001.pdf]

Emulating the MERINO randomised control trial using data from an observational cohort and trial of rapid diagnostic (BSI-FOO)

Supplementary material

Supplemental tables

Supplementary Table S1 Duration and time to receipt of emulated intervention 3
Supplementary Table S2 Baseline characteristics of patients in the MERINO trial analysis population vs. emulated trial population 4
Supplementary Table S3 Baseline characteristics of patients by emulated trial intervention, inverse probability weighted 7

Supplemental figures

Supplementary Figure S1 Flowchart 2

Supplementary Figure S1 Flowchart

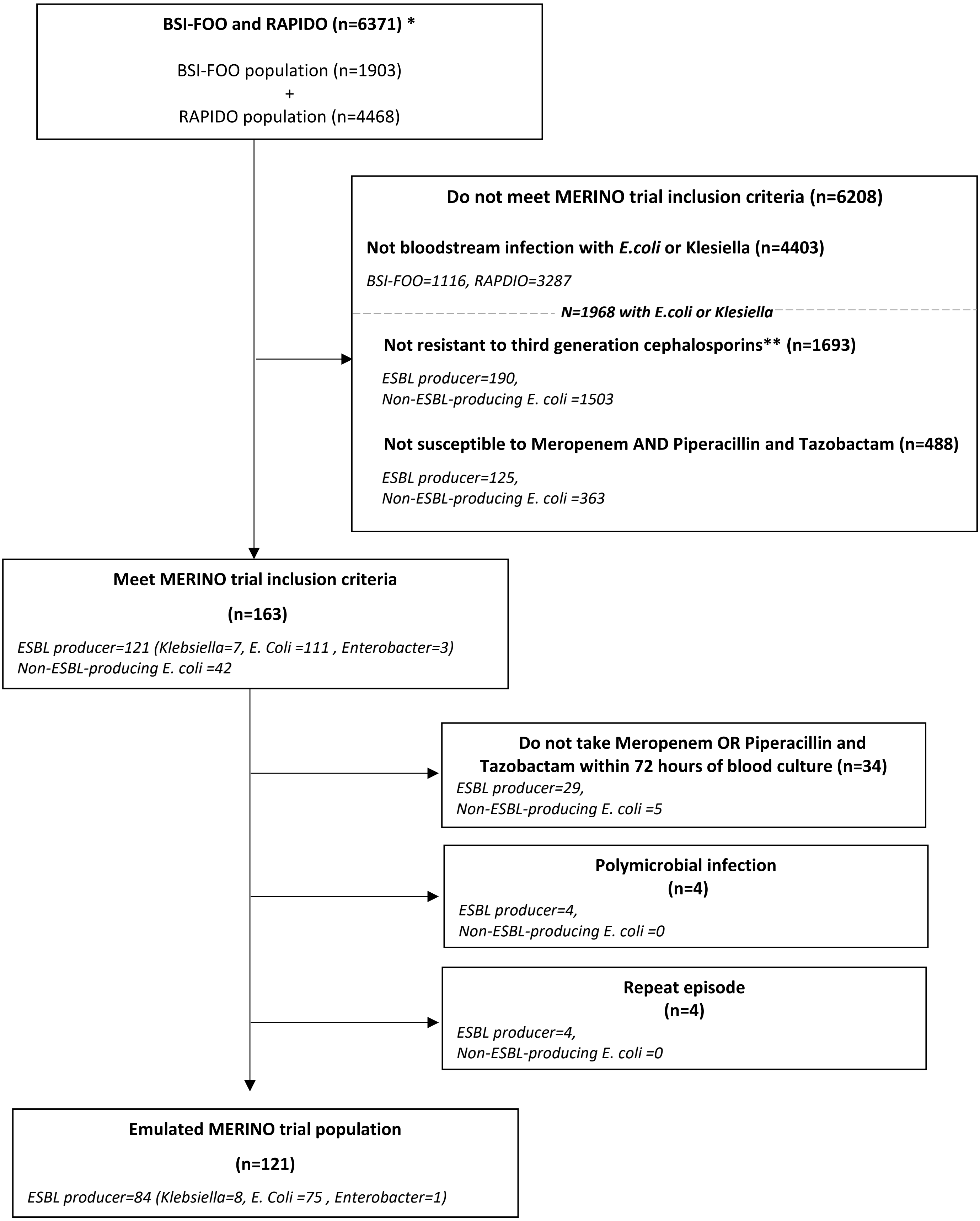

* All BSI-FOO/RAPIDO participants were ages 18 years and over and RAPIDO participant provided informed consent

** Ceftriaxone OR Cefotaxime

Supplementary Table S1 **Duration and time to receipt of emulated intervention**

	Meropenem (n=39)	Piperacillin-Tazobactam (n=82)	Overall (n=121)
	n %	n %	n %
**Time to receipt of allocated drug**			
Median hours (IQR)	38 (8, 54)	5 (0, 19)	7 (1, 32)
**Total duration of allocated drug ***			
Median days (IQR)	7 (4, 8)	3 (2, 5)	4 (2, 7)
**Duration category**			
Died within 4 days	1/39 (2.6%)	7/82 (8.5%)	8/121 (6.6%)
Intervention received <4 days	7/39 (17.9%)	37/82 (45.1%)	44/121 (36.4%)
Intervention received ≥4 days	31/39 (79.5%)	38/82 (46.3%)	69/121 (57.0%)
In combination with other active drug	4/39 (10.3%)	15/82 (18.3%)	19/121 (15.7%)
Allocated drug only	27/39 (69.2%)	23/82 (28.0%)	50/121 (41.3%)
**Crossover**			
Switch to other intervention during follow-up	1/39 (2.6%)	39/82 (47.6%)	40/121 (33.1%)

* Total duration of study drug in MERINO (Median, IQR): Meropenem = 6 days (5, 9); Piperacillin-Tazobactam = 6 days (5, 10)

Abbreviations: IQR=Interquartile range

Supplementary Table S2 **Baseline characteristics of patients in the MERINO trial analysis population vs. emulated trial population**

		MERINO trial analysis population				Emulated trial population			
		Piperacillin-Tazobactam (n=188)		Meropenem (n=191)		Piperacillin-Tazobactam (n=82)		Meropenem (n=39)	
		n	%	n	%	n	%	n	%
**Organism**									
E. coli		162/188	86.2%	166/191	86.9%	77/82	93.9%	36/39	92.3%
Klebsiella		26/188	13.8%	25/191	13.1%	5/82	6.1%	3/39	7.7%
**Stratification ***									
**E1:** E. coli, less severe		159/188	84.6%	162/191	84.8%	74/79	93.7%	32/35	91.4%
**E2:** E. coli, more severe		3/188	1.6%	3/191	1.6%	1/79	1.3%	0/35	0.0%
**K1:** Klebsiella, less severe		23/188	12.2%	25/191	13.1%	4/79	5.1%	3/35	8.6%
**K2:** Klebsiella, more severe		3/188	1.6%	1/191	0.5%	0/79	0.0%	0/35	0.0%
**Patient measures**									
Age (years)	Median (IQR)	70.0	(55.0, 78.0)	69.0	(59.0, 78.0)	74.5	(63.0, 84.0)	70.0	(54.0, 82.0)
Male		101/188	53.7%	97/191	50.8%	46/82	56.1%	15/39	38.5%
Weight (kg) [a]	Mean (SD)	67.2	18.1	69.3	19.3	70.0	15.9	72.1	24.6
**Acquisition ****									
Hospital-acquired		52/188	27.7%	46/191	24.1%	35/82	42.7%	16/39	41.0%
Health-care associated		55/188	29.3%	61/191	31.9%				
Community associated		81/188	43.1%	84/191	44.0%	47/82	57.3%	23/39	59.0%
**Source of infection**									
Bone and joint						0/82	0.0%	1/39	2.6%
Gastrointestinal system						10/82	12.2%	6/39	15.4%

Intra-abdominal infection		34/188	18.1%	28/191	14.7%				
Line infection – central venous line						1/82	1.2%	1/39	2.6%
Vascular catheter-related		3/188	1.6%	3/191	1.6%				
Lower respiratory tract						1/82	1.2%	1/39	2.6%
Reproductive tract						0/82	0.0%	1/39	2.6%
Skin and soft tissue		4/188	2.1%	1/191	0.5%	0/82	0.0%	1/39	2.6%
Surgical site infection		8/188	4.3%	4/191	2.1%	1/82	1.2%	0/39	0.0%
Systemic Infection						0/82	0.0%	1/39	2.6%
Urinary tract infection		103/188	54.8%	128/191	67.0%	46/82	56.1%	20/39	51.3%
Pneumonia		9/188	4.8%	3/191	1.6%				
Mucositis/neutropenia		12/188	6.4%	7/191	3.7%				
Musculoskeletal		1/188	0.5%	0/191	0.0%				
Other		2/188	1.1%	1/191	0.5%				
Site uncertain		12/188	6.4%	16/191	8.4%	23/82	28.0%	7/39	17.9%
**Other patient measures**									
Surgery requiring overnight stay within past 14 (MERINO) or 7 (BSI-FOO) days		19/188	10.1%	14/191	7.3%	3/82	3.7%	2/39	5.1%
ICU admission		13/188	7.0%	14/191	7.3%	6/82	7.3%	4/39	10.3%
Charlson score [b]	Median (IQR)	2.0	(1.0, 4.0)	2.0	(1.0, 4.0)	3.0	(2.0, 4.0)	4.0	(2.0, 5.0)
Pitt score [c]	Median (IQR)	1.0	(0.0, 2.0)	1.0	(0.0, 2.0)	1.0	(0.0, 2.0)	0.0	(0.0, 2.0)
Neutropenia		16/188	8.5%	9/191	4.7%	11/79	13.9%	2/38	5.3%
Urinary catheter/nephrostomy ***		51/188	27.1%	37/191	19.4%	21/59	35.6%	7/32	21.9%
Moderate-sever renal dysfunction		31/188	16.5%	30/191	15.7%	51/81	63.0%	21/37	56.8%
Diabetes		59/188	31.4%	79/191	41.4%	25/82	30.5%	10/39	25.6%

Liver disease	12/188	6.4%	18/191	9.4%	13/69	18.8%	5/31	16.1%

* Severity definition: More severe= nonurinary source and Pitt score >4. Less Severe= Urinary source, or nonurinary source and Pitt score ≤4.

** Hospital acquired if date of blood culture is >48 hours after date of admission in BSI-FOO. Data on healthcare associated infections was not collected in BSI-FOO.

*** Urinary catheter only in BSI-FOO

[a] Data missing for 57 BSI-FOO patients (14 Meropenem, 43 Piperacillin-Tazobactam)

[b] Data missing for 29 BSI-FOO patients (10 Meropenem, 19 Piperacillin-Tazobactam)

[c] Data missing for 12 BSI-FOO patients (4 Meropenem, 8 Piperacillin-Tazobactam)

Abbreviations: IQR=Interquartile range, SD= Standard deviation, ICU=Intensive care unit

Supplementary Table S3 **Baseline characteristics of patients by emulated trial intervention, inverse probability weighted**

		Meropenem (n=39) n %	Piperacillin-Tazobactam (n=82) n %
**Patient measures**			
Age	Median (IQR)	72.0 (62.0, 80.0)	73.0 (63.0, 83.0)
Male		31 (50.8%)	30 (48.5%)
Body Mass Index	Mean (SD)	27.1 (9.4)	24.7 (4.8)
**Patient medical history**			
Chemotherapy in month before date 0		1 (1.6%)	12 (20.1%)
Any tumour within last 5 years		21 (35.1%)	21 (35.6%)
Surgery requiring overnight stay within 7 days before date 0		2 (2.8%)	2 (3.1%)
Burn requiring admission within 7 days before date 0		0 (0.0%)	0 (0.0%)
Cardiac arrest within 7 days before date 0		0 (0.0%)	0 (0.0%)
Renal support within 7 days before date 0		4 (6.9%)	1 (2.4%)
Myocardial infarction within 7 days before date 0		9 (14.9%)	7 (11.3%)
**Infection severity measures**			
Temperature (°C) at time 0	Median (IQR)	38.2 (37.8, 38.8)	38.2 (37.4, 38.7)
INR	Median (IQR)	1.3 (1.2, 1.9)	1.2 (1.1, 1.4)
eGFR (mL/min/1.73m^2)	Median (IQR)	44.0 (25.0, 70.0)	50.0 (29.0, 80.0)
Neutrophil count at day 0 or closest (x10^9/L)	Median (IQR)	10.7 (6.9, 13.7)	10.7 (3.6, 15.6)

Systolic BP at day 0 or closest (mmHg)	Mean (SD)	120.8 (21.8)	118.3 (29.0)
On IV fluids at day 0		21 (34.9%)	25 (41.7%)
On ventilation at day 0		5 (8.5%)	6 (9.6%)
On vasopressor drugs at day 0		3 (5.1%)	2 (3.2%)
Systemic corticosteroids in last 24 hours		5 (8.9%)	5 (8.9%)
EWS score nearest to day 0		2.0 (2.0, 6.0)	2.0 (1.0, 3.0)
**Patient comorbidities at date 0**			
Congestive heart failure		7 (12.3%)	8 (12.8%)
Peripheral vascular disease		6 (9.1%)	7 (11.6%)
Cerebrovascular disease		17 (27.7%)	14 (23.6%)
Hemiplegia		0 (0.0%)	3 (5.2%)
Dementia		5 (9.0%)	6 (10.0%)
COPD		6 (9.5%)	9 (14.6%)
Connective tissue disease		4 (6.5%)	4 (6.6%)
Peptic ulcer disease		15 (24.3%)	4 (6.6%)
Ascites		2 (3.1%)	3 (4.3%)
Diabetes:			
None		48 (79.2%)	42 (69.0%)
Without organ damage		9 (14.4%)	14 (21.7%)
With organ damage		4 (6.5%)	6 (9.3%)
Child-Pugh score	Median (IQR)	7.0 (6.0, 8.0)	6.0 (6.0, 9.0)
Charlson score	Median (IQR)	4.0 (2.0, 5.0)	3.0 (2.0, 4.0)
Abscess at time 0		0 (0.0%)	2 (3.8%)

Infected foreign body at time 0	1 (1.9%)	0 (0.0%)
Surgical prosthesis time 0	0 (0.0%)	1 (1.2%)
**Source of infection**		
Bone and joint	2 (3.9%)	0 (0.0%)
Gastrointestinal system	12 (19.7%)	8 (13.7%)
Line infection – central venous line	1 (1.7%)	1 (1.0%)
Lower respiratory tract	1 (1.6%)	1 (1.0%)
Reproductive tract	1 (1.6%)	0 (0.0%)
Skin and soft tissue	3 (5.0%)	0 (0.0%)
Surgical site infection	0 (0.0%)	1 (1.0%)
Systemic Infection	2 (2.6%)	0 (0.0%)
Urinary tract infection	31 (51.9%)	33 (54.2%)
Site uncertain	7 (12.0%)	18 (29.1%)
**Lines and catheters**		
Central line present at time 0	8 (16.4%)	10 (23.9%)
Peripheral line present at time 0	24 (49.0%)	22 (53.4%)
Urinary catheter present at time 0	6 (11.9%)	17 (40.2%)
**Organisational factors**		
Centre:		
A	7 (11.1%)	5 (7.6%)
B	16 (26.0%)	14 (23.0%)
C	18 (29.1%)	21 (35.1%)

D	8 (13.7%)	8 (13.5%)
E	10 (16.2%)	11 (17.7%)
F	1 (1.0%)	0 (0.0%)
G	2 (3.0%)	2 (3.1%)
Ward specialty on day 0:		
Medicine	30 (50.2%)	37 (60.5%)
Critical care	4 (6.7%)	5 (8.3%)
Major surgery	21 (34.6%)	12 (20.0%)
Minor surgery	0 (0.0%)	1 (2.3%)
Other	5 (8.5%)	5 (8.9%)

Notes: Date and time 0 = date/time of sampling for blood culture

Abbreviations: IQR=Interquartile range, SD= Standard deviation, INR=International normalised ratio, eGFR=Estimated glomerular filtration rate, BP=Blood pressure, IV=Intravenous, EWS=Early warning score, COPD=Chronic obstructive pulmonary disease
